# CAMK2A supported tumor initiating cells of lung adenocarcinoma by upregulating SOX2 through EZH2 phosphorylation

**DOI:** 10.1038/s41419-020-2553-6

**Published:** 2020-06-01

**Authors:** Si-Qi Wang, Jing Liu, Jing Qin, Yun Zhu, Vicky Pui-Chi Tin, Judy Wai Ping Yam, Maria Pik Wong, Zhi-Jie Xiao

**Affiliations:** 1 Department of Pathology, The University of Hong Kong, Hong Kong, Hong Kong; 2 School of Pharmaceutical Sciences (Shenzhen), Sun Yat-sen University, Guangzhou, China; 3 Department of Pediatric Surgery, Guangzhou Institute of Pediatrics, Guangzhou Women and Children’s Medical Center, Guangzhou Medical University, Guangzhou, Guangdong China

**Keywords:** Cancer, Non-small-cell lung cancer

## Abstract

Tumor initiating cells (TIC) of lung cancer are mainly induced by stress-related plasticity. Calcium/Calmodulin dependent protein kinase II alpha (CAMK2A) is a key calcium signaling molecule activated by exogenous and endogenous stimuli with effects on multiple cell functions but little is known about its role on TIC. In human lung adenocarcinomas (AD), CAMK2A was aberrantly activated in a proportion of cases and was an independent risk factor predicting shorter survivals. Functionally, CAMK2A enhanced TIC phenotypes in vitro and in vivo. CAMK2A regulated SOX2 expression by reducing H3K27me3 and EZH2 occupancy at SOX2 regulatory regions, leading to its epigenetic de-repression with functional consequences. Further, CAMK2A caused kinase-dependent phosphorylation of EZH2 at T487 with suppression of EZH2 activity. Together, the data demonstrated the CAMK2A-EZH2-SOX2 axis on TIC regulation. This study provided phenotypic and mechanistic evidence for the TIC supportive role of CAMK2A, implicating a novel predictive and therapeutic target for lung cancer.

## Introduction

Lung cancer has the highest mortality of all cancers despite treatment advances such as targeted therapy. Adenocarcinoma (AD) is the commonest histo-morphological type of primary lung carcinoma in both male and female regardless of smoking history. One important reason for treatment failure is functional heterogeneity of different cell subpopulations in a cancer with variable drug sensitivity. While some cancers such as leukaemia comprise an indigenous stem cell-like subset capable of self-renewal and cancer perpetuation, such a population is not well delineated in human lung cancers. On the other hand, it is believed under environmental stress, cancer cells can acquire “stemness” properties through plasticity to maintain cancer growth and become phenotypic tumor initiating cells (TIC). TIC are present in genetically similar or distinct cell clones, thus, targeting TIC offers a treatment strategy that complements cytotoxic chemotherapy and targeted therapy^1,2^.

Molecular mechanisms that might be involved in inducing or sustaining TIC have been keenly investigated as potential treatment targets. Ca^2+^ is a second messenger that integrates signals from intrinsic and extrinsic stimulations such as growth factors, cytokines, oxidative stress, etc. It mediates diverse cell functions in a tissue- and context-dependent manner, including cancer cell proliferation, invasion, metastasis, angiogenesis, and pro-survival redox recalibration^3–5^. Multiple approaches targeting upstream calcium regulators such as Ca^2+^ channels, pumps, and transporters have been investigated, but much less attention has been devoted to effector pathways.

The CAMK2 multifunctional serine/threonine kinase is a key calcium signaling effector activated by a cytosolic Ca^2+^ surge, or through the redox-sensitive Cys280/Met281 residues leading to autophosphorylation of the catalytic domain^6–8^. There are four subunits, CAMK2A, 2B, 2D, and 2G which are distributed in different tissues and serve different functions. In this study, we investigated the possible role and mechanism of the CAMK2A subunit in mediating TIC properties of lung AD. We observed lung AD with high-level expression of CAMK2A were associated with shorter survivals than those with low expression. The tumorsphere TIC subset showed higher expression of CAMK2A compared with non-TIC. Functionally, CAMK2A supported TIC phenotypes including self-renewal, tumorigenecity, resistance to cytotoxic and targeted drug treatment through direct phosphorylation of EZH2 T487 and epigenetic de-repression of SOX2. This novel finding suggested CAMK2A could be a treatment approach that aims to target TIC in lung AD.

## Results

### CAMK2A overexpression correlated with adverse patient survivals

**Fig. 1:**
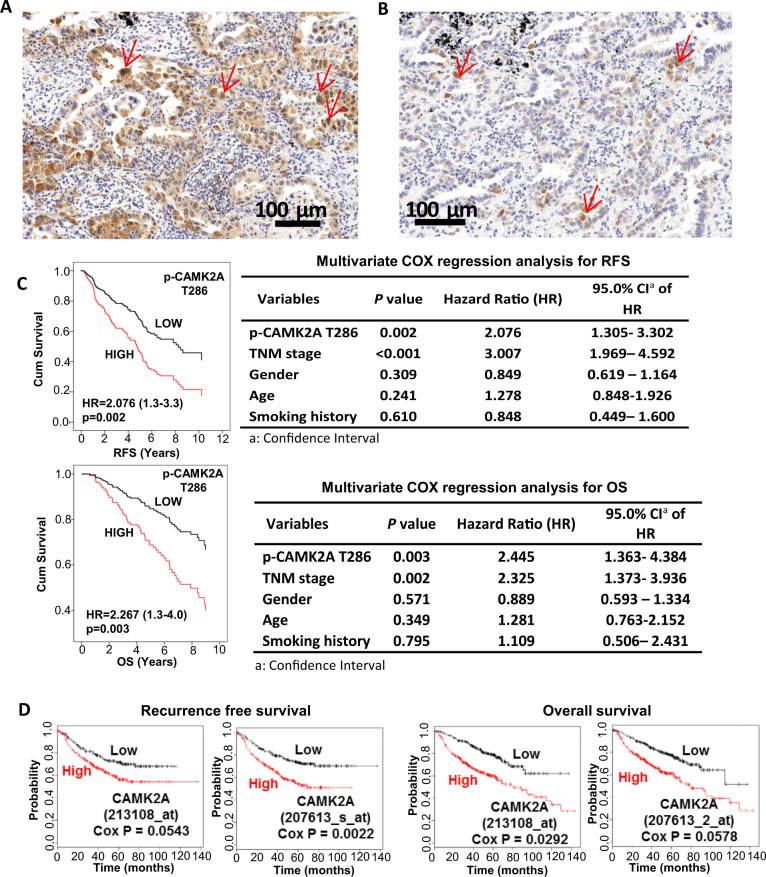
Correlation of activated CAMK2A expression in lung AD with patient survivals. **a**, **b** Immunohistochemical (IHC) evaluation of p-CAMK2A T286 expression in lung adenocarcinoma. **a** High-level expression, showing higher frequency of tumor cells staining positively for cytoplasmic and nuclear signals (arrows). **b** Low-level expression, showing fewer positively stained tumor nuclei (arrows) in the same unit area as shown in Fig. 1b. **c** COX regression analysis for recurrence-free survival (RFS) and overall survival (OS) stratified by p-CAMK2A T286 levels. Survival curves were generated by SPSS 18.0. **d** COX regression analysis of CAMK2A effects on RFS and OS using public expression array data (www.kmplot.com/lung).

Immunohistochemistry (IHC) for activated CAMK2A (p-CAMK2A T286) was performed on 199 clinical lung AD. Expression was observed in tumor cell cytoplasm and nuclei with non-uniform intensities among different tumor cells within the same region. This observation implied CAMK2A activation was not clonal in nature but influenced by cell-to-cell variation in microenvironmental conditions. Tumors with high-level expression (37.7%) comprised those with higher proportions of tumor cells showing intense nuclear staining, while tumors with low level staining (62.3%) were those that showed no or few cells with weak nuclear staining (Fig. 1a, b). Cox regression analysis showed tumors with higher expression significantly correlated with shorter recurrence free (RFS, *p* = 0.002) and cancer-specific overall survivals (OS, *p* = 0.003) (Fig. 1c). Both survival parameters also correlated with the pathological tumor stage (RFS, *p* ≤ 0.001; OS, *p* = 0.002) but not with other factors including patients’ age, gender, and smoking history. Furthermore, IHC for total CAMK2A proteins showed high-level expression in 40.8% of 377 AD, which featured more frequent and intense staining in tumor cell cytoplasm only (Supplementary Fig. 1a, b). Kaplan Meier survival analysis showed tumors with high total CAMK2A expression were significantly correlated with shorter recurrence free survival (RFS, *p* = 0.012) (Supplementary Fig. 1c). Further, expression of CAMK2A and p-CAMK2A analyzed by correlation analysis showed significant and positive correlation (Pearson *R* = 0.145, *p* = 0.021) (Supplementary Fig. 1d). Survival analysis was also performed using lung cancer data from the public expression microarray databases (www.kmplot.com/lung). CAMK2A was the only subunit that showed significant and consistent association with survivals for all probe sets (Fig. 1d), while other subunits showed either reverse correlation (CAMK2D) or inconsistent results between probe sets (CAMK2B, CAMK2G) (Supplementary Fig. 2a, b).

Together, the findings indicated a prognostic role of CAMK2A and p-CAMK2A in lung AD survival.

### CAMK2A supported tumorigenesis in lung AD

**Fig. 2:**
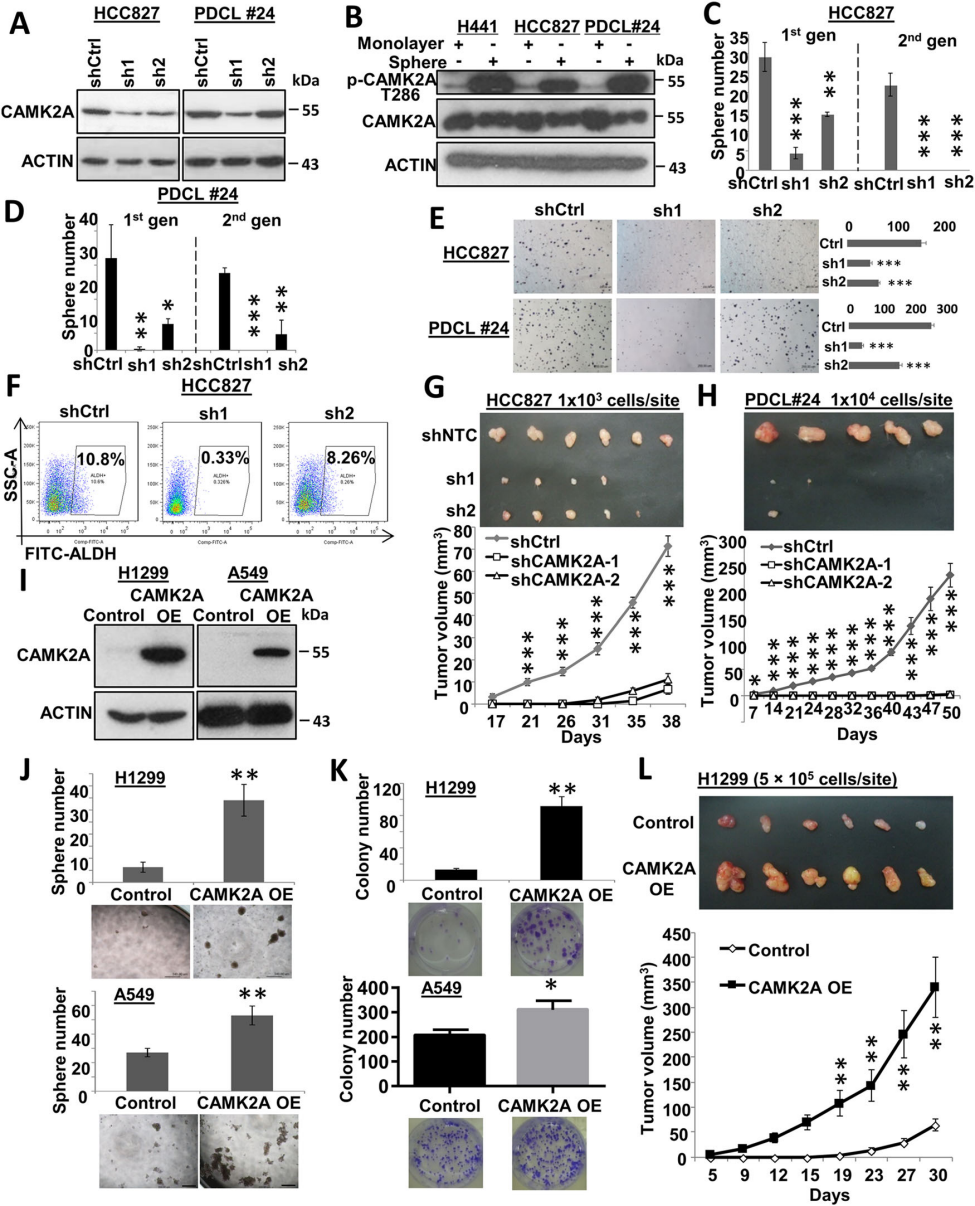
Effects of CAMK2A on TIC properties in lung AD cells. **a** Western blot of shRNA-mediated CAMK2A KD in HCC827 and PDCL#24 cells. **b** Activated CAMK2A (p-T286) relative to total CAMK2A expression was increased in spheres compared with cell monolayers of H441, HCC827 and PDCL#24. **c**, **d** Sphere formation of both cell lines with CAMK2A KD were inhibited for two generations. **e** Colony formation of CAMK2A KD cells were decreased in both cell lines. **f** Flow cytometry of HCC827 with CAMK2A KD showed reduction of ALDH-expressing cells. **g**, **h** Tumor sizes and growth curves of xenografts with CAMK2A KD from HCC827 (**g**) and PDCL#24 (**h**) were suppressed compared with controls (shNTC). **i** Western blot of CAMK2A OE in H1299 and A549 cells. **j**, **k** Sphere formation (**j**) and colony formation (**k**) of H1299 and A549 with CAMK2A OE were increased. **l** Tumor growth of H1299-derived xenografts with CAMK2A OE was facilitated. *p < 0.05; **p < 0.01; ***p < 0.001 compared with control. Data represented mean ± SD.

Multiple lung AD cell lines with various CAMK2A expression levels were manipulated to model their in vitro and in vivo roles on TIC phenotypes (Supplementary Fig. 3a). Using two independent lentiviral shRNAs (sh1, sh2, respectively), CAMK2A was stably knocked down (KD) in HCC827 and PDCL#24 resulting in suppressed p-CAMK2A T286, consistent with effective KD (Fig. 2a and Supplementary Fig. 3b). The expression of activated CAMK2A was significantly higher in the tumorspheres compared with non-stem cell surrogates growing in monolayers (Fig. 2b). Upon KD, functional changes were observed, e.g., tumorspheres were significantly reduced or completely suppressed for two consecutive generations, with the magnitudes of change corresponding with the degrees of CAMK2A suppression (Fig. 2c, d). Colony formation was also reduced by CAMK2A suppression (Fig. 2e). Flow cytometry showed the TIC subset marked by ALDH^+^ was reduced (Fig. 2f). In vivo, decreasing inoculation doses of 5 × 10^3^ and 1 × 10^3^ HCC827 CAMK2A-KD cells grown as monolayers, respectively, gave rise to significantly suppressed tumor growth rates (Fig. 2g and Supplementary Fig. 3c). Similarly, significantly attenuated tumorigenesis was observed for xenografts raised from 1 × 10^4^ of PDCL#24 cells grown as monolayers (Fig. 2h).

Conversely, in H1299 and A549 cells that showed low basal CAMK2A levels, CAMK2A was overexpressed which resulted in upregulation of both CAMK2A and p-CAMK2A (Fig. 2i and Supplementary Fig. 3d). CAMK2A overexpression (OE) led to significantly increased tumorspheres (Fig. 2j), colony formation (Fig. 2k) and xenograft growth rates raised from monolayer cultures of H1299 (Fig. 2l and Supplementary Fig. 3e).

### CAMK2A increased resistance to cytotoxic and target therapy in lung AD

**Fig. 3:**
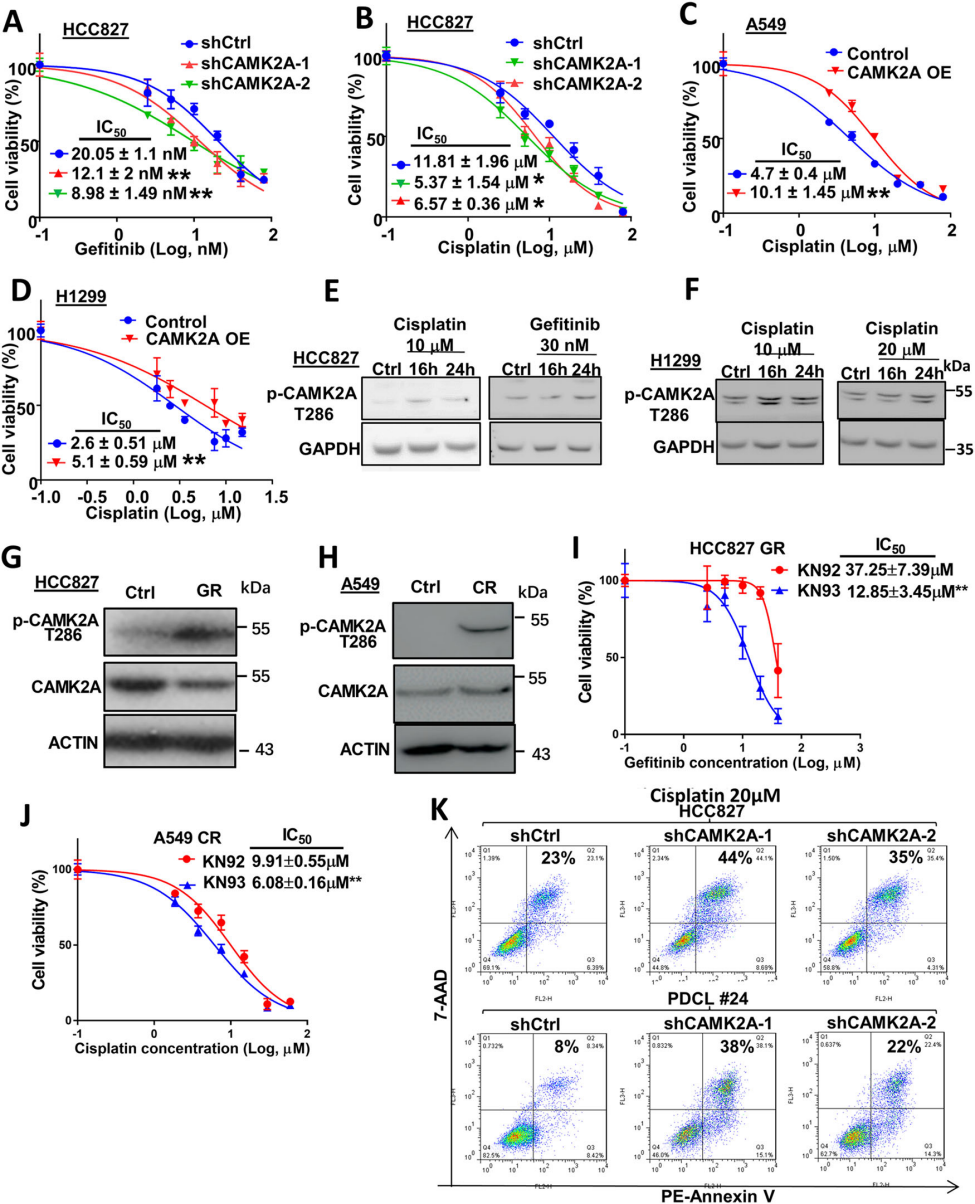
Effects of CAMK2A on responses of lung AD cells to target therapy and cytotoxic drugs. **a**, **b** Cell viability of HCC827 with CAMK2A KD showed increased sensitivity to gefitinib (**a**) and cisplatin (**b**). **c**, **d** Cell viability of A549 (**c**) and H1299 (**d**) with CAMK2A OE showed increased resistance to cisplatin. **e**, **f** Western blot showed that short term treatment of gefitinib and cisplatin increased p-CAMK2A level in HCC827 (**e**) and H1299 cells (**f**). **g**, **h** Western blot of gefitinib-resistant HCC827 (GR) (**g**) and cisplatin-resistant A549 (CR) cells (**h**) showed increased p-CAMK2A T286 relative to total CAMK2A compared with parental control cells. **i**, **j** Cell viability of HCC827 GR cells (**i**) and A549 CR cells (**j**) under CAMK2A inhibition by KN93 showed sensitization to gefitinib and cisplatin, respectively. **k** Flow cytometry analysis of cisplatin treated HCC827 and PDCL#24 cells with CAMK2A KD showed increased apoptosis. *p < 0.05; **p < 0.01, compared with control, mean ± SD was presented.

CAMK2A-inhibited cells were tested for viability under a dose range of targeted therapy and cisplatin chemotherapy. The IC_50_ of gefitinib, a targeted therapy for HCC827 harboring *EGFR exon19* deletion, was significantly reduced from the control level of 20.05 to 12.1 nM and 8.98 nM, respectively (*p* < 0.01) (Fig. 3a). Sensitivity to cisplatin chemotherapy was also significantly enhanced, shown by the reduced IC_50_ from 11.81 to 5.37 µM and 6.57 µM, respectively (*p* < 0.05) (Fig. 3b). Conversely, for the *KRAS* mutant cell line A549, overexpressing CAMK2A significantly increased resistance, raising IC_50_ from 4.7 to 10.1 µM (*p* < 0.01) (Fig. 3c), and for H1299, from 2.6 to 5.1 µM (*p* < 0.01) (Fig. 3d). Short- term drug treatments of HCC827 and H1299 were able to activate CAMK2A, shown s increased p-CAMK2A T286 compared with control levels after 16 or 24 h, respectively, (Fig. 3e, f). Together, results implicated elevated levels of CAMK2A increased lung cancer cell resistance to both target therapy and cisplatin chemotherapy.

The role of CAMK2A in mediating chronic drug resistance was further investigated. Stable gefitinib-resistant (GR) cells of HCC827 and cisplatin-resistant (CR) cells of A549 were generated by chronic exposure to increasing dosages of the respective drugs (up to 10 µM of gefitinib and 5 µM of cisplatin, respectively) (Supplementary Fig. 4a, b). Both HCC827 GR and A549 CR cells exhibited enhanced TIC phenotypes, demonstrated by the increased proportion of ALDH^high^/CD44^high^ population of TIC reported in our previous studies^9,10^, and enhanced sphere forming ability (Supplementary Fig. 4c, d). The resistant cells showed enhanced CAMK2A phosphorylation (Fig. 3g, h). The CAMK2 inhibitor KN93 was employed to study the effects of pharmacologic CAMK2A inhibition on chronic drug resistance, and its inactive chemical derivative KN92 was used as the control. Because KN93 and KN92 demonstrate CAMK2 kinase-independent actions such as calcium or potassium channels blockade, the use of KN93/92 pairs would enable more specific evaluation of CAMK2-mediated actions^11^. To determine the treatment dose of KN93 for our experiments, cell viability was assessed with escalating doses of KN93 and KN92. As shown in Supplementary Fig. 4e, almost all A549 cells were viable under up to 5 µM of KN93, which effectively suppressed the expression level of p-CAMK2A T286 in HCC827 cells (Supplementary Fig. 4f). Co-treatment of the resistant HCC827 GR cells with the CAMK2 inhibitor KN93 at 5 µM induced a significantly lower IC_50_ (12.85 µM) compared with its control analogue KN92 (IC_50_ 37.25 µM) (*p* < 0.05) (Fig. 3i). Likewise, the parental HCC827 cells also showed a lower IC_50_ for KN93 (14.4 µM) than KN92 (26.8 µM) (Supplementary Fig. 4g). Thus, the data implicated CAMK2 inhibition by KN93 sensitized the parental and GR cells of HCC827 to gefitinib treatment. For A549 CR cells, significantly reduced IC_50_ from 9.91 µM (for KN92) to 6.08 µM (for KN93) (*p* < 0.05) indicating sensitization to cisplatin by CAMK2 treatment was observed. On the other hand, KN93 treatment did not significantly alter the cisplatin IC_50_ of A549 parental cells (Supplementary Fig. 4h). This insignificant effect of KN93 on A549 cells could be due to the low basal CAMK2A level in A549 parental cells. Additionally, the effects of CAMK2A-KD on cisplatin-treated cells was studied using flow cytometry, where apoptotic fractions were increased by 1.5–1.9 folds for HCC827, and by 2.7–4.7 folds for PDCL#24 (Fig. 3k). Together, the data showed chronic drug treatment activated CAMK2A in lung cancer cells leading to increased resistance to both EGFR target therapy and cisplatin chemotherapy.

### CAMK2A’s role in self renewal and tumorigenesis was mediated through SOX2

**Fig. 4:**
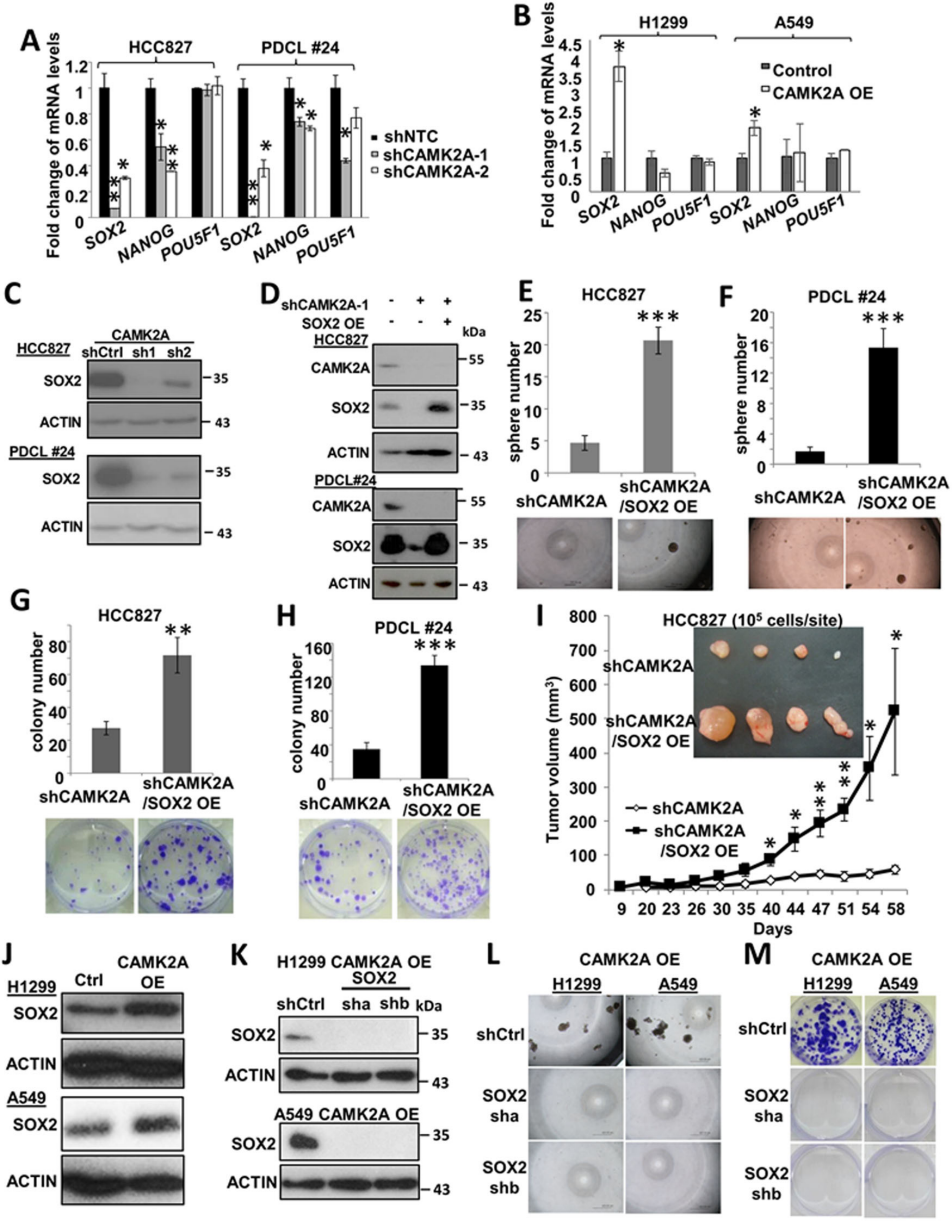
SOX2-mediated effects of CAMK2A TIC in lung AD. **a**, **b** Quantitative PCR of *SOX2*, *NANOG* and *POU5F1* transcripts in HCC827 and PDCL#24 cells with CAMK2A KD (**a**), and in H1299 and A549 cells with CAMK2A OE (**b**). **c** Western blot showing SOX2 decrease in HCC827 and PDCL#24 cells with CAMK2A KD. **d** Western blot showing SOX2 changes in HCC827 and PDCL#24 cells with CAMK2A KD and SOX2 OE. **e**–**h** Overexpression of SOX2 increased sphere formation (**e**, **f**) and colony formation (**g**, **h**) in CAMK2A-downregulated HCC827 and PDCL#24 cells. **i** SOX2 overexpression enhanced tumorigenicity in HCC827 shCAMK2A-1-derived xenografts. *n* = 4. **j** Expression of SOX2 in CAMK2A-overexpressed H1299 and A549 cells by western blot. **k** Downregulation of SOX2 in CAMK2A-overexpressed H1299 and A549 cells was confirmed by western blot. **l**, **m** Downregulation of SOX2 abrogated CAMK2A’s tumor initiating properties in sphere formation (**l**) and colony formation (**m**) in H1299 and A549 CAMK2A-overexpressed cells. *p < 0.05; **p < 0.01; ***p < 0.001, compared with control, mean ± SD was presented.

To investigate the mechanisms of CAMK2A on TIC support, its effects on pluripotency genes expressions were studied. Of the three key factors *SOX2, NANOG* and *POU5F1*, CAMK2A manipulation produced the most consistent and largest magnitude of change on *SOX2* transcripts, while *NANOG* and *POU5F1* showed either inconsistent or insignificant changes (Fig. 4a, b). Results of *SOX2* were further confirmed at the protein level, confirming CAMK2A mediated SOX2 up-regulation (Fig. 4c).

Functionally, over-expressing SOX2 was able to restore tumorspheres and colony formation of CAMK2A-deficient cells of HCC827 and PDCL#24 (Fig. 4d–h), as well as xenograft initiation ability of HCC827 (Fig. 4i). In H1299 and A549 cells with CAMK2A-OE, respectively, SOX2 was upregulated (Fig. 4j), and its suppression abrogated the above in vitro cancer phenotypes (Fig. 4k–m).

### CAMK2A regulated SOX2 through EZH2 suppression

**Fig. 5:**
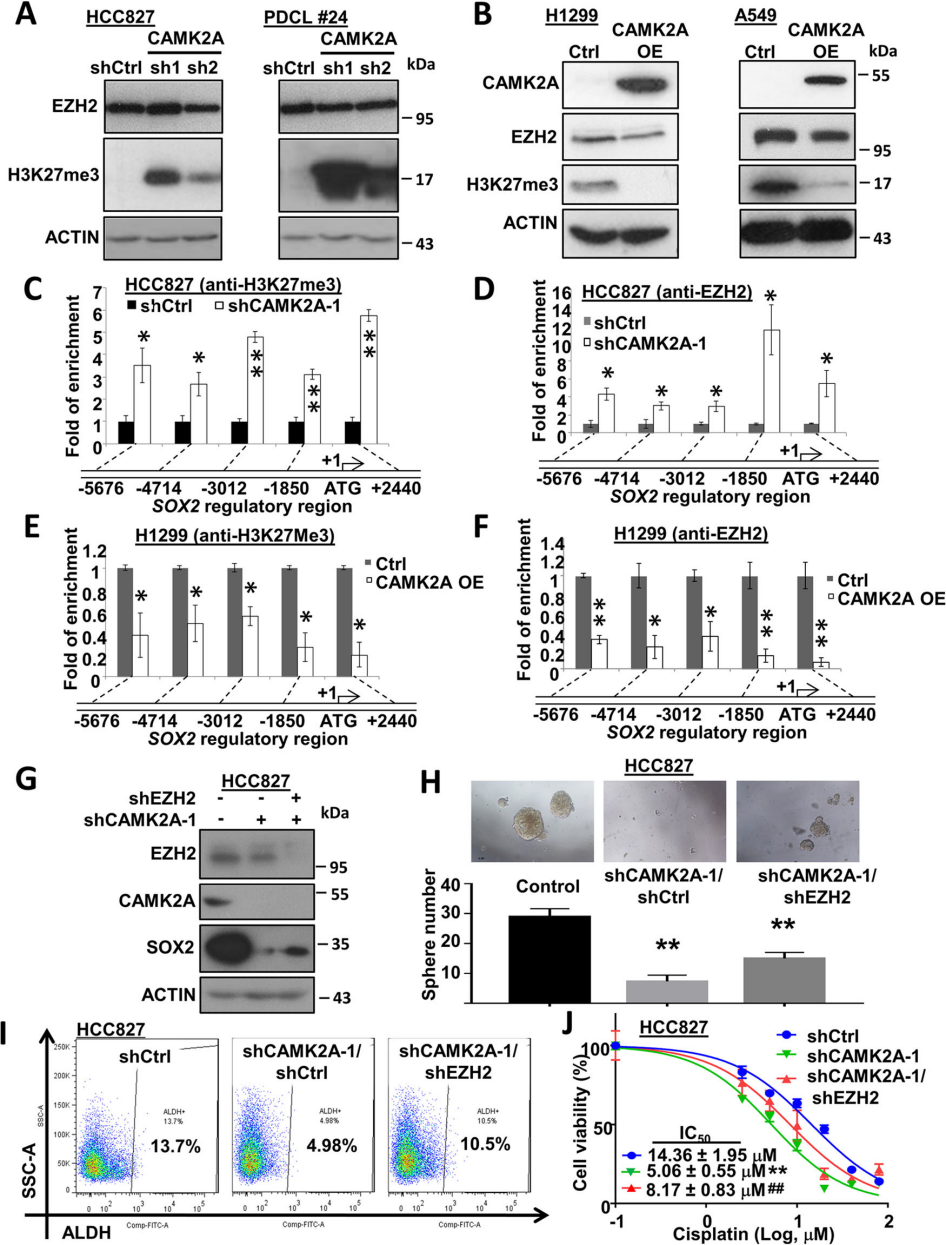
CAMK2A enhanced SOX2 expression through decreasing EZH2 mediated H3K27me3 in the regulatory region of *SOX2*. **a**, **b** Expression of EZH2 and H3K27me3 in CAMK2A-downregulated HCC827 and PDCL #24 cells (**a**) as well as CAMK2A-overexpressed H1299 and A549 cells (**b**) by western blot. **c**, **d** Chromatin immunoprecipitation (ChIP) assay coupled with qPCR (ChIP-qPCR) analysis revealed the relative enrichment of H3K27me3 (**c**) and EZH2 (**d**) on *SOX2* regulatory region in CAMK2A-downregulated HCC827 cells. **e**, **f** ChIP-qPCR analysis revealed the relative decrease of H3K27me3 (**e**) and EZH2 (**f**) on *SOX2* regulatory region in CAMK2A-overexpressed H1299 cells. **g**–**j** Downregulation of EZH2 rescued the inhibitory effects of CAMK2A knockdown on SOX2 expression (**g**), sphere formation (**h**), ALDH^+^ population (**i**) and cisplatin sensitivity (**j**) in HCC827 cells. *p < 0.05; **p < 0.01; ***p < 0.001, compared with control, ^##^*p* < 0.01 compared with shCAMK2A, mean ± SD was presented.

Epigenetic regulation is the most important machinery for modifying stemness activities. To investigate the role of CAMK2A in this regard, effects of CAMK2A manipulation on several histone marks were studied. Compared with controls, CAMK2A-KD in both HCC827 and PDCL#24 markedly increased the repressive histone mark H3K27me3 but those on EZH2, which is known to mediate H3K27me3 trimethylation, were not significantly changed (Fig. 5a). Conversely, with CAMK2A-OE, H3K27me3 was prominently suppressed but EZH2 was only slightly reduced (Fig. 5b). Evaluation of additional histones showed inconsistent changes for H3K4me3, H3K27ac and H3K9me2 in CAMK2A manipulated cells (Supplementary Fig. 5a–c). To further clarify the relation, we next evaluated the accessibility of *SOX2* regulatory regions by studying H3K27me3 and EZH2 occupancy at multiple *SOX2* regulatory loci using ChIP-qPCR. In HCC827 with CAMK2A-KD, precipitation of *SOX2* sequences were increased 3–5 folds by anti-H3K27me3 (Fig. 5c), while they were increased by 4–14 folds using anti-EZH2 (Fig. 5d), respectively. The data implicated suppression of CAMK2A led to reduced SOX2 expression through increased H3K27me3 and EZH2 binding. Reciprocally, in H1299 with CAMK2A-OE, binding of H3K27me3 and EZH2 to *SOX2* regulatory sequences were reduced (Fig. 5e, f).

The above postulation was further verified by functional studies. While stable CAMK2A-KD markedly suppressed SOX2 expression and tumorspheres formation, additional EZH2-KD led to partial recovery of SOX2 levels and significantly restored tumorspheres (Fig. 5g, h). As we previously reported^10^, SOX2 could mediate reactive oxygen species (ROS) regulation in lung TIC through ALDH. Accordingly, EZH2-KD rescued ALDH activities from 4.98 to 10.5% (Fig. 5i). Further, while CAMK2A-KD sensitized HCC827 cells to cisplatin with reduced IC_50_ from 14.36 to 5.06 µM, additional EZH2-KD partly reverted this to 8.17 µM (*p* < 0.01) (Fig. 5j). Similarly, addition of the EZH2 inhibitor GSK126 to CAMK2A-KD cells could also reverse the cisplatin sensitizing effect (Supplementary Fig. 6a). Also, while knockdown of CAMK2A sensitized HCC827 cells to gefitinib, additional EZH2-KD partially reverted this effect (Supplementary Fig. 6b). Taken together, the data suggested CAMK2A mediated in vitro cancer phenotypes and drug resistance through reducing EZH2 and release of SOX2 from epigenetic repression. However, the exact molecular mechanism needed clarification.

### CAMK2A reduced EZH2 activity by direct phosphorylation at EZH2 T487

**Fig. 6:**
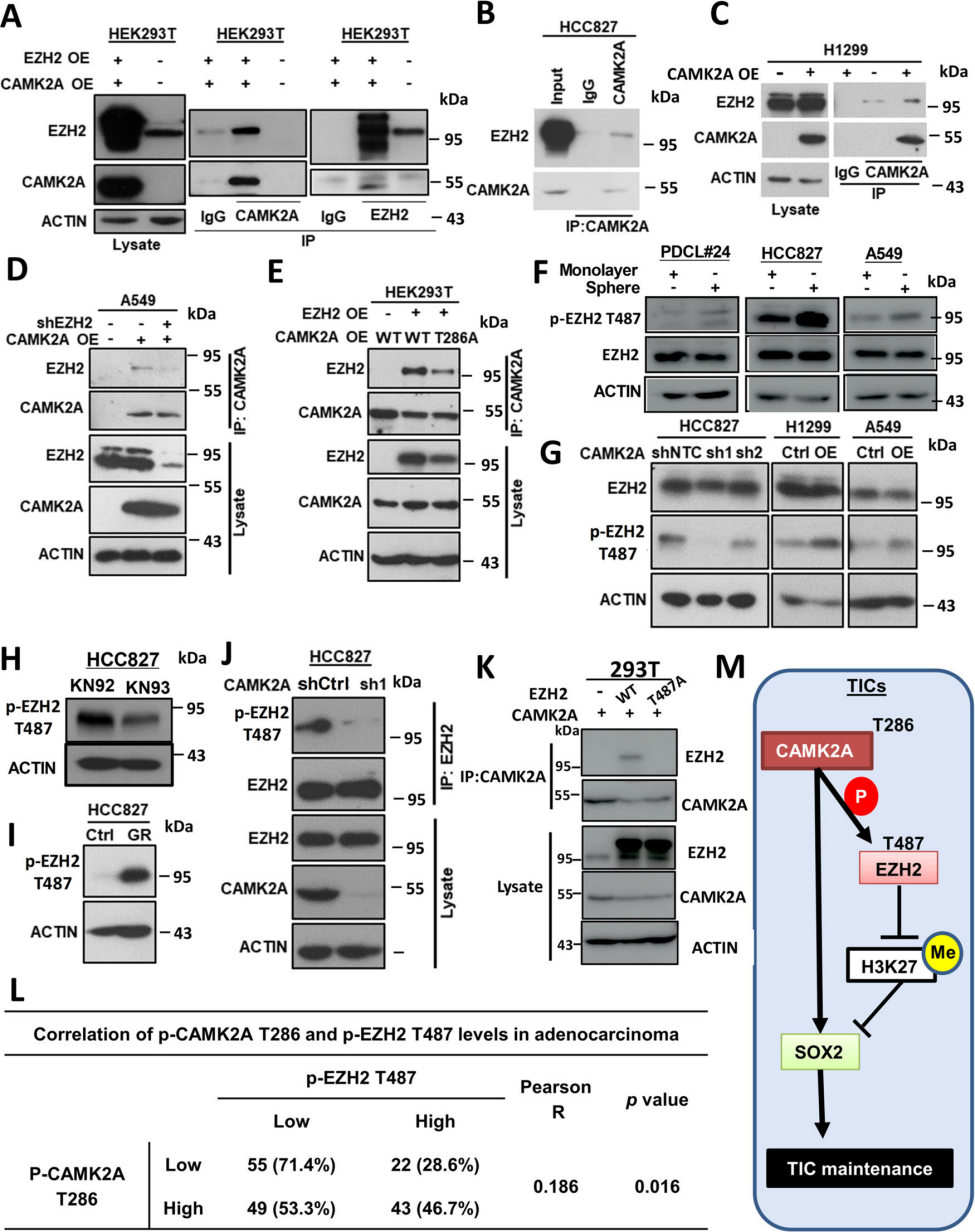
CAMK2A phosphorylated EZH2 at T487. **a** Co-immunoprecipitation (CoIP) in HEK293T cells with dual overexpression of CAMK2A and EZH2, either using CAMK2A or EZH2 as bait. **b** CoIP of EZH2 in HCC827 cells using CAMK2A as bait. **c** CoIP of EZH2 in H1299 CAMK2A-OE cells. **d** CoIP of EZH2 in A549 CAMK2A-OE cells with or without EZH2 knock-down using CAMK2A anti-body. **e** CoIP of EZH2 in HEK293T cells with overexpression of EZH2, CAMK2A WT or CAMK2A T286A using CAMK2A as bait. **f** Expression of total EZH2 and p-EZH2 T487 in monolayer and spheres from PDCL#24, HCC827, and A549 cells. **g** Expression of total EZH2 and p-EZH2 T487 in HCC827, H1299 and A549 cells with CAMK2A knock-down or overexpression by immunoblot. **h** Expression of p-EZH2 T487 in HCC827 cells treated with KN92/KN93 by immunoblot. **i** Expression of p-EZH2 T487 in HCC827 GR cells by immunoblot. **j** Immunoprecipitated p-EZH2 T487 level by EZH2 in HCC827 cells with or without CAMK2A knock-down. **k** CoIP of EZH2 in HEK293T cells with overexpression of CAMK2A, EZH2 WT or EZH2 T487A using CAMK2A as bait. **j** Correlation analysis between p-CAMK2A T286 and p-EZH2 T487 expression in 169 primary resected lung AD by immunohistochemistry. **k** Schematic representation depicting supportive effect of CAMK2A on TIC maintenance through EZH2/H3K27me3/SOX2 pathway.

To study whether CAMK2A directly interacted with EZH2, CoIP was performed using either molecule as the bait alternately. Both reactions yielded a much higher level of the other molecule compared with the isotype matched controls, respectively (Fig. 6a). In HCC827, endogenous EZH2 could also be immunoprecipitated using anti-CAMK2A (Fig. 6b). In tumorspheres grown from HCC827 and A549 cells, higher levels of EZH2 could be precipitated by anti-CAMK2A compared with their corresponding monolayers (Supplementary Fig. 7a). From H1299 with stable CAMK2A-OE, anti-CAMK2A precipitated a higher EZH2 level compared with control (Fig. 6c), while in A549, additional EZH2-KD led to correspondingly reduced EZH2 precipitant (Fig. 6d). Thus, using multiple cell lines with or without forced CAMK2A expression, direct binding between CAMK2A and EZH2 was demonstrated.

At the functional level, ectopic expression of the kinase-deficient CAMK2A (T286A) in HEK293T led to reduced EZH2 precipitant compared with CAMK2A wild-type cells (Fig. 6e). Together with the functional effects of EZH2 KD (Fig. 5h–j), our findings supported CAMK2A might mediate TIC supportive roles through directly binding and phosphorylating EZH2, resulting in reduced H3K27me3 occupancy and *SOX2* de-repression.

Previous results showed clearly CAMK2A suppressed H3K27me3 levels but its effects on total EZH2 was elusive. On the other hand, phosphorylation of EZH2 at T487 is known to increase its proteasomal degradation and hence reduce H3K27me3 trimethylation. We therefore investigated whether CAMK2A could mediate EZH2 T487 phosphorylation. Indeed, p-EZH2 T487 was upregulated in tumorspheres compared with their corresponding monolayer isolated from PDCL#24, HCC827 and A549 cells (Fig. 6f). Furthermore, WB showed p-EZH2 T487 was correspondingly increased or reduced on CAMK2A up- or downregulation, respectively (Fig. 6g), and CAMK2A inhibition by KN93 could suppress p-EZH2 T487 level (Fig. 6h). In HCC827 GR cells with CAMK2A activation, higher level of p-EZH2 T487 was observed compared with parental cells (Fig. 6i). While CAMK2A overexpression resulted in increased p-EZH2 T487 and SOX2 expression but decreased H3K27me3 levels, ectopic expression of mutant CAMK2A T286A did not increase p-EZH2 T487 and SOX2 levels and retained H3K27me3 level compared with control cells (Supplementary Fig. 7b). Further validation was shown by EZH2 immunoprecipitation, where HCC827 with CAMK2A KD yielded reduced p-EZH2 T487 precipitants (Fig. 6j), and A549 with CAMK2A OE yielded increased precipitants (Supplementary Fig. 7c). While overexpression of EZH2 resulted in higher level of EZH2 precipitated by CAMK2A antibody, the phosphor-deficient mutant form of EZH2 (EZH2 T487A) failed to be precipitated by CAMK2A (Fig. 6k).

Lastly, to interrogate the clinical relevance of CAMK2A kinase activity on p-EZH2 T487, IHC was performed on 169 primary resected lung AD which showed a modest correlation between activated p-CAMK2A T286 and p-EZH2 T487 (*p* = 0.016) (Fig. 6l), implicating in vivo interaction between the two proteins.

## Discussion

CAMK2 comprises four subunits with distinct linker regions and identical functional domains. Early studies on CAMK2 mostly employed pharmacological inhibition and the exact subunits and their roles involved in different functions were not studied. The subunits are distributed in different tissues and conduct context-dependent functions. CAMK2A is normally expressed in the brain facilitating learning and long-term memory. A few reports have suggested CAMK2A plays a role in cancers of the breast, colon, and stomach^12–14^, but involvement in lung or in TIC maintenance has not been reported. CAMK2B is expressed in neural tissues and CAMK2D in heart and skeletal muscles with little information on cancers. To study whether CAMK2D might play a TIC supporting role in lung cancer, we knocked down CAMK2D by siRNA in HCC827 cells, and the effect on TIC phenotypes was studied (Supplementary Fig. 8a). Results showed CAMK2D knockdown did not alter either the expression of pluripotency genes (Supplementary Fig. 8b) or drug sensitivity of HCC827 cells (Supplementary Fig. 8c, d), indicating that CAMK2D maybe not involved in lung TIC regulation. On the other hand, CAMK2G which is ubiquitously expressed has been shown to support cancers through activating transcription factors such as AKT1, CREB, CDK1/2. Chai et al. reported CAMK2G could support lung tumor through affecting stem-like traits, and suggested these were mediated through NF*k*B activation; however, whether SOX2 was involved was not addressed^15^. Gu et al. showed in chronic myeloid leukaemia, TIC were suppressed by the herbal drug berbamine and docking of berbamine traced it to CAMK2G, but no details of the mediating pathway were provided^16^. While microarray data quoted in this study showed a more consistent survival significance of CAMK2A than CAMK2G in lung cancer, more studies would help to clarify their respective roles.

The mediation of TIC in cancer perpetuation and treatment resistance has been shown in many cancers. In this and previous studies, we have shown among different pluripotency factors, SOX2 is the one most consistently upregulated with the highest magnitude of change in lung TIC surrogates such as tumorspheres and the ALDH^high^/CD44^high^ fraction^9,10^. Stem cells are believed to be carriers of cumulative somatic mutations during genomic and clonal evolution, thus, being a stem cell mediator active in upper aerodigestive tract organogenesis and postnatal tissue regeneration^17,18^, SOX2’s support of tumorigenesis is not surprising. Clinical evidence of its importance in lung cancer is further exemplified by *SOX2* amplification in squamous cell and small cell lung carcinomas, and its over-expression in TIC of AD^19–21^. Furthermore, SOX2 mediates drug resistance and renewal of myeloma, skin and bladder cancers^22,23^. Thus, we surmise pathways that regulate SOX2 expression in lung AD could be a TIC vulnerability.

Our data showed CAMK2A was a regulator of SOX2. While manipulation of CAMK2A expression in cancer cells induced corresponding functional changes consistent with its TIC supportive role including self-renewal, xenograft tumorigenecity at low cell doses, and resistance to targeted or cytotoxic therapy, silencing *SOX2* abrogated these effects. Further, cancer cells with induced drug resistance showed increased levels of activated CAMK2A, and co-treatment with its pharmacological inhibitor restored relative sensitivity.

Phenotypic reprogramming of stem cells is mainly mediated through epigenetic mechanisms. EZH2 is one of the known methyl transferases producing H3K27me3 which is most often regulated by transcription factors, tumor suppressor microRNA and cancer-associated non-coding RNA^24^. In our study, we observed CAMK2A manipulation did not cause consistent or marked variations in EZH2 levels, but changes of H3K27me3 levels and occupancy at *SOX2* regulatory regions were in line with our postulated effects of CAMK2A. Since EZH2 is a known kinase substrate, we considered whether CAMK2A produced such changes through EZH2 phosphorylation. Subsequently, using residue-specific EZH2 antibodies and overexpression of a kinase-deficient CAMK2A, we showed CAMK2A directly phosphorylated EZH2 at T487, which could reduce EZH2 activity either by its dissociation from the polycomb repressive complex 2 (PRC2)^25^, or by protein destabilization and proteasomal degradation^26^. The role of EZH2 in different cancers is complex and gene-dependent. It is oncogenic in many cancers through repression of tumor suppressor genes and derepression of oncogenes. In lung cancer, both roles have been reported, e.g., as a tumor suppressor illustrated by tumor enhancement via EZH2 depletion in a *KRAS*^*G12D*^ primary mouse lung AD model^27^, and as an oncogenic mediator illustrated by the correlation between high EZH2 protein levels with poor survival of human lung AD^28^. For our cohort, we found correlated expressions of p-EZH2 T487 and p-CAMK2A T286 (*p* = 0.016) in human lung AD. Ultimately, results from directly targeting CAMK2A in clinical studies would be required to confirm the role of CAMK2A on EZH2 phosphorylation.

In the CAMK2A-EZH2-SOX2 pathway (Fig. 6m), CAMK2A is the only factor specifically expressed in tumor but not normal lung, and thus might be a relatively safe treatment target compared with non-specifically inhibiting EZH2 and SOX2. Various CAMK2 inhibitors have been developed for other conditions such as cardiac arrhythmia and heart failure. Whether lung cancer treatment could benefit from repurposing such drugs, or from new formulations that can prevent neuronal off-target effects is worth exploring^11^.

Together with our previous report on the TIC supportive role of another calcium signaling effector, NFATc2, which is a transcription factor capable of *SOX2* upregulation through 3′ enhancer binding resulting in ROS attenuation through ALDH^10^, our studies illustrate the growing recognition of the involvement of calcium signaling in cancer biology.

## Materials and methods

### Human lung cancers and IHC

Primary lung cancer tissues from ethnic Chinese were collected in Queen Mary Hospital with informed consent after approval by HKU/HAHKWC Ethics Board. Tumor diagnosis and staging were assessed by a qualified pathologist (MPW). At least two tumor cores from each case were selected and assembled into tissue microarray blocks (TMA). IHC was performed according to routine procedures after antigen retrieval by scientific microwave at 95 °C for 15 min in pH 8.0 EDTA. p-CAMK2A T286 (1:100, Cell Signaling), total CAMK2A (1:250, Abcam) and p-EZH2 T487 (1:500, Abcam) primary antibodies were incubated overnight at 4 °C, followed by secondary antibodies conjugated with polymer-linked HRP (DAKO, Agilent) for 30 min at room temperature.

Expression levels of p-CAMK2A T286 and p-EZH2 T487 were scored according to the extent and intensity of nuclear staining in the tumor cells while expression in the cytoplasm, stromal or inflammatory cells was excluded from evaluation. Expression of total CAMK2A was evaluated based on cytoplasmic staining only. Staining intensities were scored as 1, 2, or 3 according to whether staining was absent/weak, moderate, or strong, respectively. The staining extent was graded as 1, 2, or 3 according to whether expression was observed in scattered individual cells, aggregates of 5 or more but <100 cells, or sheets of ≥100 cells. The products of the 2 grades were then computed, and cases with scores of 4 and above were counted as high-level expression.

### Cell lines

Patient derived cell lines #24 (PDCL#24) raised from resected lung cancers and only 1^st^ to 10^th^ passage cells were used for study. Other established human NSCLC cell lines were obtained from ATCC. Cells were maintained in RPMI-1640 (Invitrogen, Carlsbad, CA) with 10% FBS. For generation of stable drug resistant cell lines (A549 CR and HCC827 GR cells), A549 and HCC827 cells were cultured with stepwise increase of cisplatin and gefitinib respectively, over a period of 3 months and around 10 passages to allow the cells to adapt to cisplatin or gefitinib toxicity. The stable drug-resistant cells were cultured in drug free medium for 2 weeks before the bioassays. All procured cell lines used in this study were authenticated using the AmpFlSTR® Identifiler® PCR Amplification Kit for short tandem repeat profiling according to the manufacturer’s instruction (Thermo Fisher Scientific, Waltham, MA).

### Flow cytometry

ALDH activity was analyzed by the Aldefluor kit (Stem Cell Technologies) using fluorescence activated cell sorting (FACS) Canto II (BD Biosciences) as described^9^.

### Sphere formation assay and in vitro passage

Five hundred cells were cultured in an ultra-low plate (Costar) with TIC medium for 14 days. Harvested spheres were enzyme dissociated and seeded again for the second passage as previously described^10^.

### Colony formation and soft agar colony formation

Five hundred cells were cultured in 6-well plates for 2 weeks for colony formation assay. For soft agar colony formation, 25,000 cells were mixed with 0.35% agarose layered on plates that were pre-coated with a layer of 0.5% agarose in RMPI-1640 medium with 10%FBS for 4 weeks.

### Drug sensitivity and apoptosis assays

Drug sensitivity assay was performed by Thiazolyl Blue Tetrazolium Bromide (MTT) test and apoptosis was quantified by Annexin V-PE and 7-AAD staining.

### Gene expression analysis

Gene mRNA expression was analyzed by quantitative RT-PCR (qPCR) (7900HT, Applied Biosystems, Carlsbad, CA) and SYBR green (Qiagen, Hilden, Germany) detection. Expressions of *RPL13A* and *beta-2-microglobulin* (*B2M*) were averaged and used as an internal control. Primers were listed in Supplementary Table 1.

### In vivo tumorigenicity

All animal experiments were approved and performed according to guidelines by the Animal Ethics Committee, the University of Hong Kong. Briefly, cells grown as monolayer are harvested and mixed with an equal volume of matrigel (BD Pharmingen). Cell suspension were injected subcutaneously at the back of 6-week old severe combined immunodeficiency (SCID) mice. Tumors were measured using digital vernier calipers, with tumor volume calculated using the formula [sagittal dimension (mm) × cross dimension (mm)^2^]/2.

### Plasmids for stable lentiviral knockdown and overexpression

ShRNA targeting human CAMK2A and EZH2 were from Sigma-Aldrich (St Louis, MO). ShRNA targeting SOX2 a and b (26353, 26352), the negative control vector pLKO.1-puro (1864), the envelope vector pMD2.G (12259) and packaging vector psPAX2 (12260) were from Addgene (Cambrige, MA; http://www.addgene.org). Human full length CAMK2A was cloned into PCDH-CMV-MCS-EF1-COPGFP vector (SBI, Mountain View, CA) for stable CAMK2A overexpression. pSin-EF2-SOX2-Pur (16577, Addgene) was used for stable SOX2 overexpression. Lentiviral production and infection were performed as previously described^10^. Stable cells were either selected by treating puromycin (2 µg/ml, Sigma-Aldrich) or by FACS using BD Aria (BD Biosciences).

### Chromatin immunoprecipitation (ChIP) assay

ChIP assay was performed using Magna ChIP^TM^ A kit (Millipore, Billerica, MA) according to manufacturer’s instructions. Quantification of antibody-bound region was done by qPCR performed with ABI Prism 7900HT (Applied Biosystems, Carlsbad, CA). Primers of SOX2 regulatory regions derived from public epigenetic maps spanning −5676 nt to +2460 nt were listed in the Supplementary Table S1. Rabbit IgG was used as negative control in the assay.

### Co-immunoprecipitation (CoIP) and immunoblot

For CoIP, 1 mg of cell lysate was incubated with 3 µg of anti-EZH2 (Cell Signaling, MA) or anti-CAMK2A (Santa Cruz, CA) antibody, respectively, with gentle rotation at 4 °C for 4 h. Twenty-five microliters of protein-G Mag Sepharose Xtra (GE Healthcare) were washed five times with NETN buffer. The antibody complex was incubated with protein-G beads overnight at 4 °C. Beads were washed with NETN buffer for five times. The eluted immunoprecipitants were subjected to SDS-PAGE analysis. Primary antibodies against SOX2, EZH2, H3K27me3 and ACTIN (Cell Signaling, MA) were incubated overnight followed by anti-rabbit secondary antibody incubation. Target proteins on the membrane were visualized on X-ray films using ECL Plus Western Blotting Detection Reagents (Amersham, Buckinghamshire, UK).

### Statistics

All results were derived from at least three independent experiments. Data were analyzed by SPSS (version 18.0; SPSS Inc., Chicago, IL, USA) or GraphPad Prism 7.0 and shown as mean ± standard deviations (SD). For continuous variables, differences between groups were analyzed by Student’s *t* test. Association between p-CAMK2A T286 expression and survivals were analyzed by COX regression analysis. Association between CAMK2A expression and survivals were analyzed by Log rank test. Correlation between CAMK2A and p-CAMK2A T286, and p-CAMK2A T286 and p-EZH2 T487 levels were analyzed by *χ*^2^-test. A two-tailed *p* value of <0.05 was considered as the threshold for statistical significance.

## Supplementary information

Supplementary table 1

Supplementary Figure 1

Supplementary Figure 2

Supplementary Figure 3

Supplementary Figure 4

Supplementary Figure 5

Supplementary Figure 6

Supplementary Figure 7

Supplementary Figure 8

Supplementary figure legends

## References

[1] Kreso A, Dick JE (2014). Evolution of the cancer stem cell model. Cell Stem Cell.

[2] Saygin C, Matei D, Majeti R, Reizes O, Lathia JD (2019). Targeting cancer stemness in the clinic: from hype to hope. Cell Stem Cell.

[3] Roderick HL, Cook SJ (2008). Ca^2+^ signalling checkpoints in cancer: remodelling Ca^2+^ for cancer cell proliferation and survival. Nat. Rev. Cancer.

[4] Stewart TA, Yapa KT, Monteith GR (2014). Altered calcium signaling in cancer cells. Biochimica et. Biophysica Acta.

[5] Hempel N, Trebak M (2017). Crosstalk between calcium and reactive oxygen species signaling in cancer. Cell Calcium.

[6] Hunter T, Schulman H (2005). CaMKII structure–an elegant design. Cell.

[7] Chao LH (2011). A mechanism for tunable autoinhibition in the structure of a human Ca2+/calmodulin- dependent kinase II holoenzyme. Cell.

[8] Erickson JR (2014). Mechanisms of CaMKII activation in the heart. Front. Pharmacol..

[9] Liu J (2013). Lung cancer tumorigenicity and drug resistance are maintained through ALDHhiCD44hi tumor initiating cells. Oncotarget.

[10] Xiao, Z. J. et al. NFATc2 enhances tumor-initiating phenotypes through the NFATc2/SOX2/ALDH axis in lung adenocarcinoma. *eLife* **6**, 10.7554/eLife.26733 (2017). 10.7554/eLife.26733 PMC5570574 28737489

[11] Pellicena P, Schulman H (2014). CaMKII inhibitors: from research tools to therapeutic agents. Front. Pharmacol..

[12] Liu Z, Han G, Cao Y, Wang Y, Gong H (2014). Calcium/calmodulindependent protein kinase II enhances metastasis of human gastric cancer by upregulating nuclear factorkappaB and Aktmediated matrix metalloproteinase9 production. Mol. Med. Rep..

[13] Chi M (2016). Phosphorylation of calcium/calmodulin-stimulated protein kinase II at T286 enhances invasion and migration of human breast cancer cells. Sci. Rep..

[14] Chen W (2017). Ca^(2+)^/calmodulin-dependent protein kinase II regulates colon cancer proliferation and migration via ERK1/2 and p38 pathways. World J. Gastroenterol..

[15] Chai S (2015). Ca^2+^/calmodulin-dependent protein kinase IIgamma enhances stem-like traits and tumorigenicity of lung cancer cells. Oncotarget.

[16] Gu Y (2012). CaMKII gamma, a critical regulator of CML stem/progenitor cells, is a target of the natural product berbamine. Blood.

[17] Sarkar A, Hochedlinger K (2013). The Sox family of transcription factors: versatile regulators of stem and progenitor cell fate. Cell Stem Cell.

[18] Gontan C (2008). Sox2 is important for two crucial processes in lung development: branching morphogenesis and epithelial cell differentiation. Dev. Biol..

[19] Karachaliou N, Rosell R, Viteri S (2013). The role of SOX2 in small cell lung cancer, lung adenocarcinoma and squamous cell carcinoma of the lung. Transl. Lung Cancer Res..

[20] Roudi R (2016). Evidence for embryonic stem-like signature and epithelial-mesenchymal transition features in the spheroid cells derived from lung adenocarcinoma. Tumour Biol..

[21] Nakatsugawa M (2011). SOX2 is overexpressed in stem-like cells of human lung adenocarcinoma and augments the tumorigenicity. Lab. Investig..

[22] Siegle JM (2014). SOX2 is a cancer-specific regulator of tumour initiating potential in cutaneous squamous cell carcinoma. Nat. Commun..

[23] Chen X (2019). Long noncoding RNA LBCS inhibits self-renewal and chemoresistance of bladder cancer stem cells through epigenetic silencing of SOX2. Clin. Cancer Res..

[24] Yamaguchi H, Hung MC (2014). Regulation and role of EZH2 in cancer. Cancer Res. Treat..

[25] Wei Y (2011). CDK1-dependent phosphorylation of EZH2 suppresses methylation of H3K27 and promotes osteogenic differentiation of human mesenchymal stem cells. Nat. Cell Biol..

[26] Wu SC, Zhang Y (2011). Cyclin-dependent kinase 1 (CDK1)-mediated phosphorylation of enhancer of zeste 2 (Ezh2) regulates its stability. J. Biol. Chem..

[27] Wang Y (2017). Ezh2 acts as a tumor suppressor in Kras-driven lung adenocarcinoma. Int. J. Biol. Sci..

[28] Behrens C (2013). EZH2 protein expression associates with the early pathogenesis, tumor progression, and prognosis of non-small cell lung carcinoma. Clin. Cancer Res..

