# Supplementary material for: CAMK2A supported tumor initiating cells of lung adenocarcinoma by upregulating SOX2 through EZH2 phosphorylation
Source: Cell Death Dis. 2020 Jun 1;11(6):410. doi: 10.1038/s41419-020-2553-6 (PMC7264342; doi:10.1038/s41419-020-2553-6)
Supplement: Supplementary file 1 — Supplementary table 1 [file 41419_2020_2553_MOESM1_ESM.docx]

**Supplementary Table S1. List of qPCR primers and ChIP assay primers**

| Gene | Primer sequences |
| --- | --- |
| *NANOG* | F: AAGGTCCCGGTCAAGAAACAG  R: CTTCTGCGTCACACCATTGC |
| *SOX2* | F: GCCGAGTGGAAACTTTTGTCG  R: GGCAGCGTGTACTTATCCTTCT |
| *POU5F1* | F: GGCAACCTGGAGAATTTGTT  R: GTGCATAGTCGCTGCTTGAT |
| *ALDH1A1* | F: ATGCTTCCGAGAGGGGGCGA  R: CCCAACCTGCACAGTAGCGCA |
| *GAPDH* | F: GAGTCAACGGATTTGGTCGTAT  R: ATGGGTGGAATCATATTGGAAC |
| *B2M* | F: AGGCTATCCAGCGTACTCCA  R: GGCATCTTCAAACCTCCAT |
| *SOX2-*Promoter Region 1 | F: TGCATTCCTTAGGTTTCAAGG |
|  | R: GCACCTTGCCTTTAATTTCC |
| *SOX2-*Promoter Region 2 | F: CACTGCAGTATGATTGCTGCT  R: GCCAGATGTGGTCAAATGAA |
| *SOX2-*Promoter Region 3 | F: AAGCAGACCGATGCATACAC  R: GGAGGTTAGACCATGGGAAA |
| *SOX2-*Promoter Region 4 | F: GCATTGAGCGCCTACCTATT  R: CGGGTTAGAGGAGGATGAGA |
| *SOX2-*Promoter Region 5 | F: TTGAGCCAAATCAGAACCAG  R: TTGCAATTATTCTGATACAAGAAGC |
| *GAPDH-*CHIP-control | F: TACTAGCGGTTTTACGGGCG |
|  | R: TCGAACAGGAGGAGCAGAGAGCGA |
