# Supplementary figures and images for: CAMK2A supported tumor initiating cells of lung adenocarcinoma by upregulating SOX2 through EZH2 phosphorylation
Source: Cell Death Dis. 2020 Jun 1;11(6):410. doi: 10.1038/s41419-020-2553-6 (PMC7264342; doi:10.1038/s41419-020-2553-6)

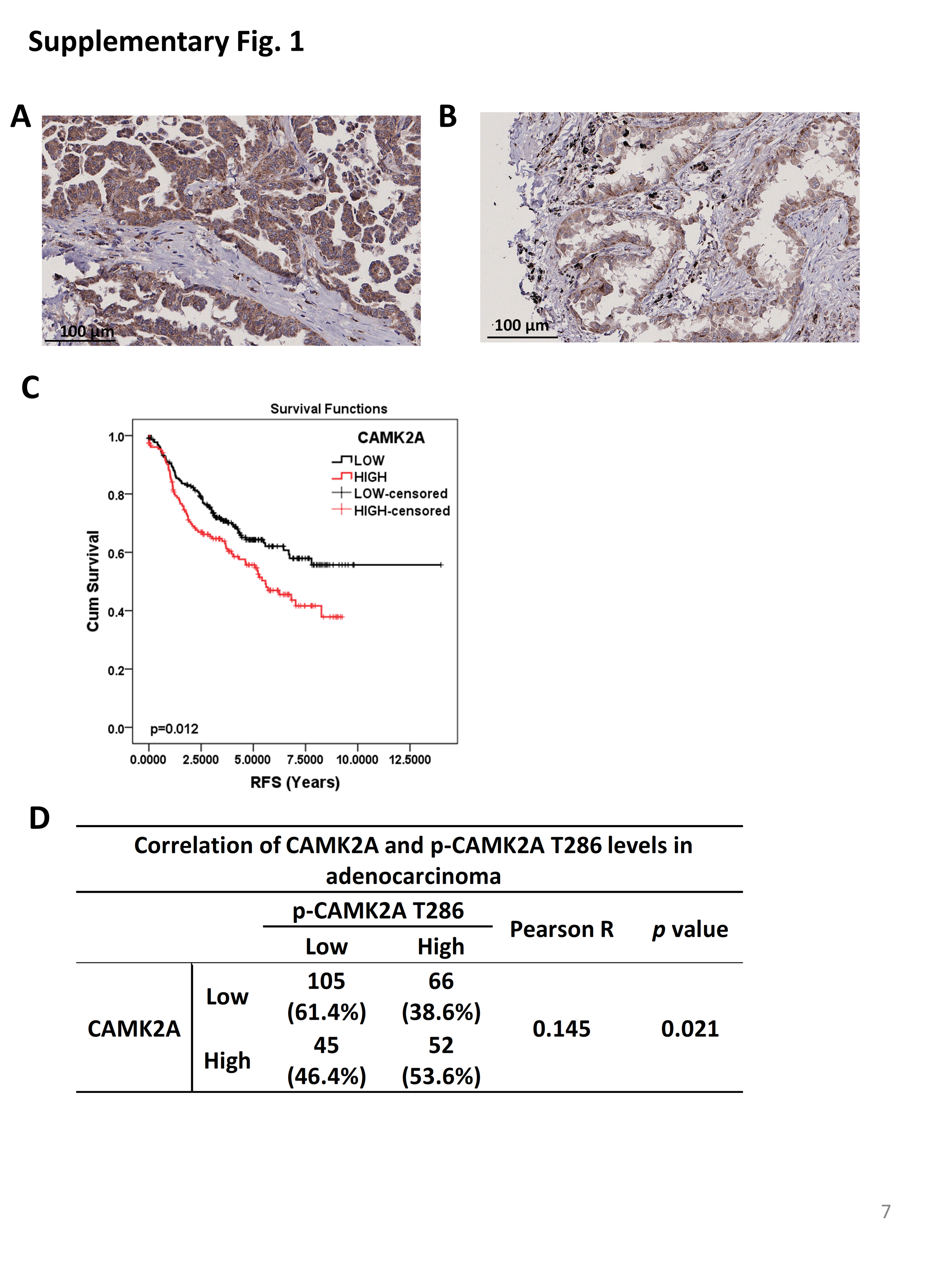

Supplement: Supplementary file 2 — Supplementary Figure 1 [file 41419_2020_2553_MOESM2_ESM.tif]

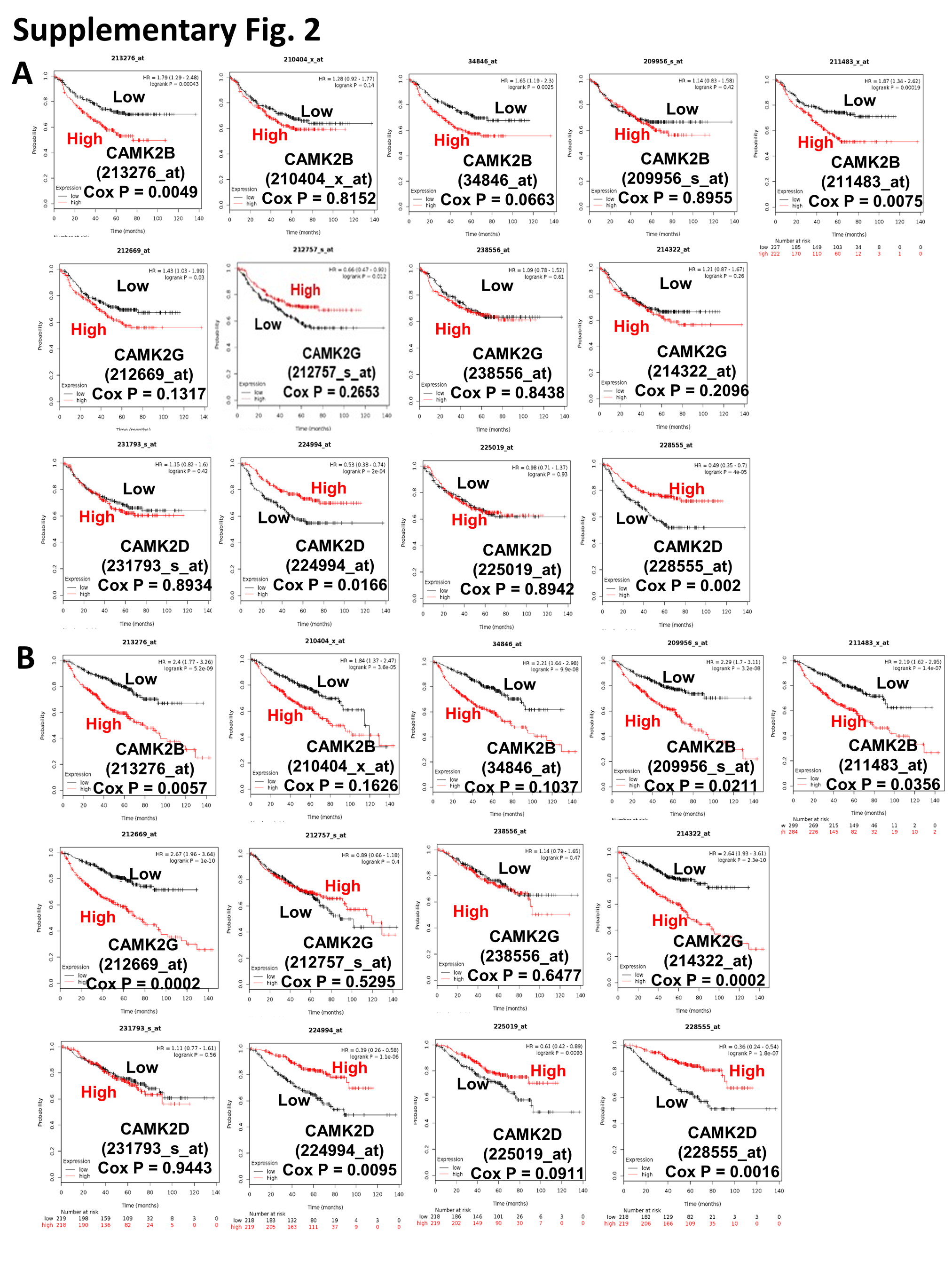

Supplement: Supplementary file 3 — Supplementary Figure 2 [file 41419_2020_2553_MOESM3_ESM.tif]

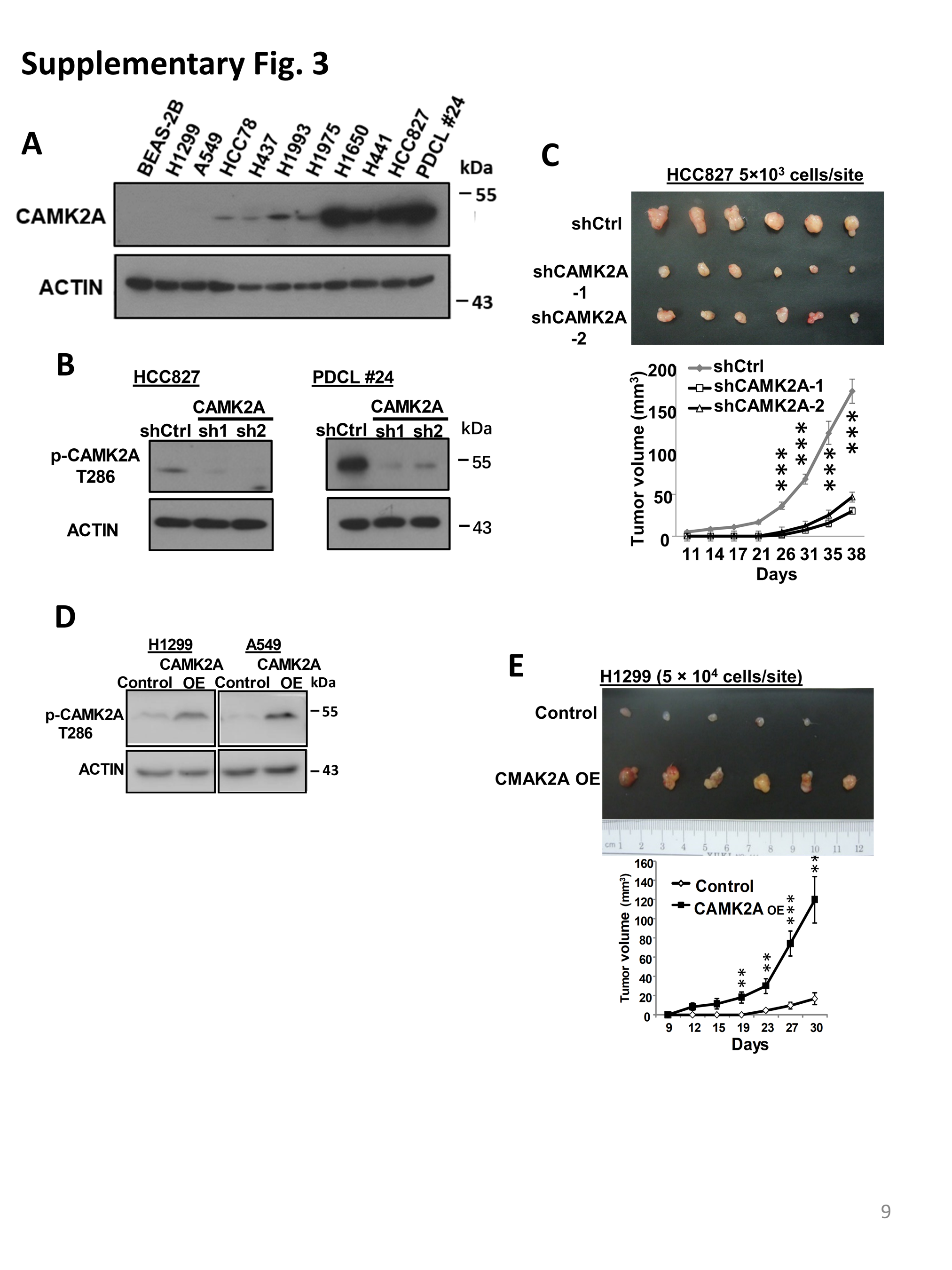

Supplement: Supplementary file 4 — Supplementary Figure 3 [file 41419_2020_2553_MOESM4_ESM.tif]

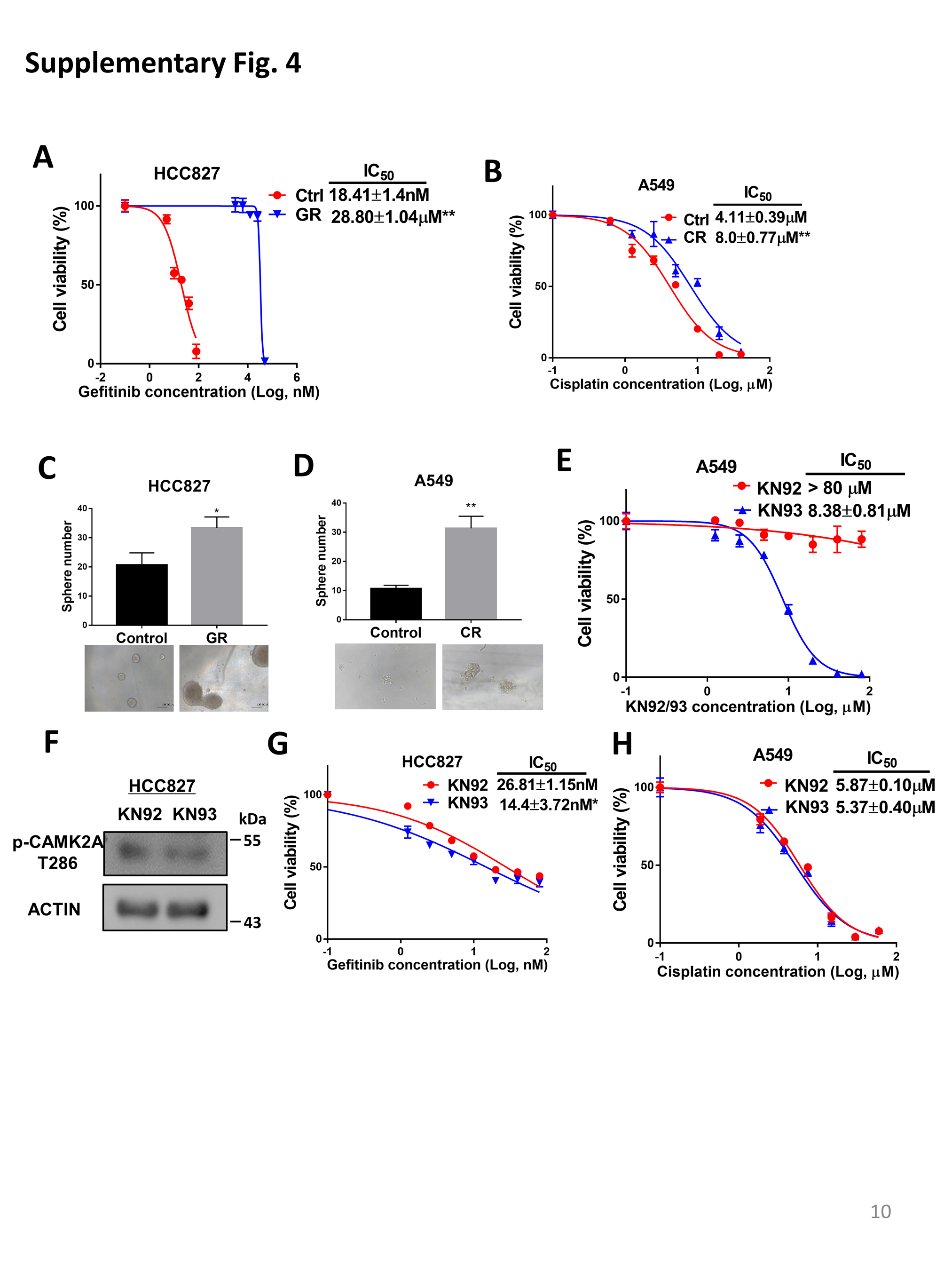

Supplement: Supplementary file 5 — Supplementary Figure 4 [file 41419_2020_2553_MOESM5_ESM.tif]

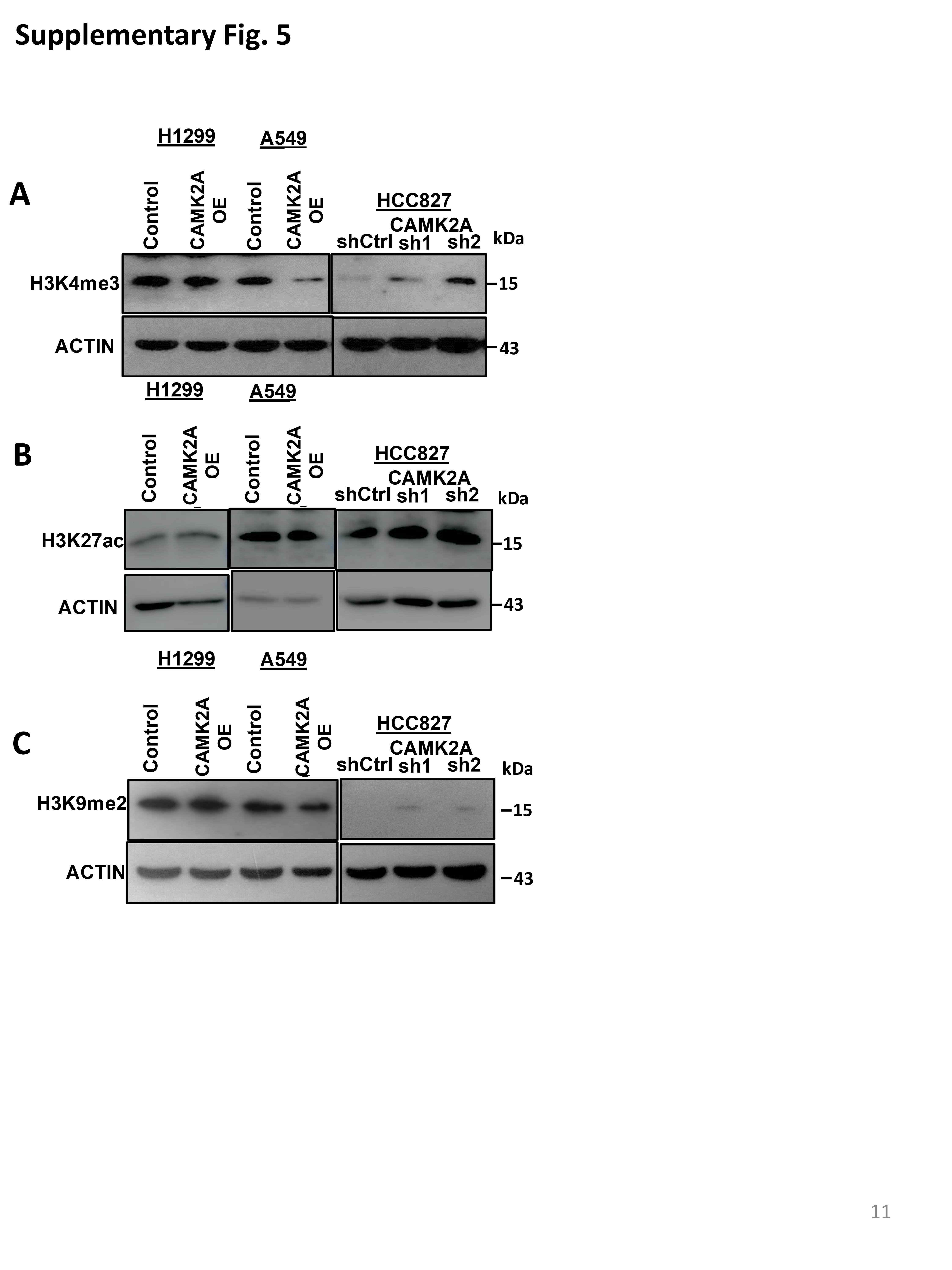

Supplement: Supplementary file 6 — Supplementary Figure 5 [file 41419_2020_2553_MOESM6_ESM.tif]

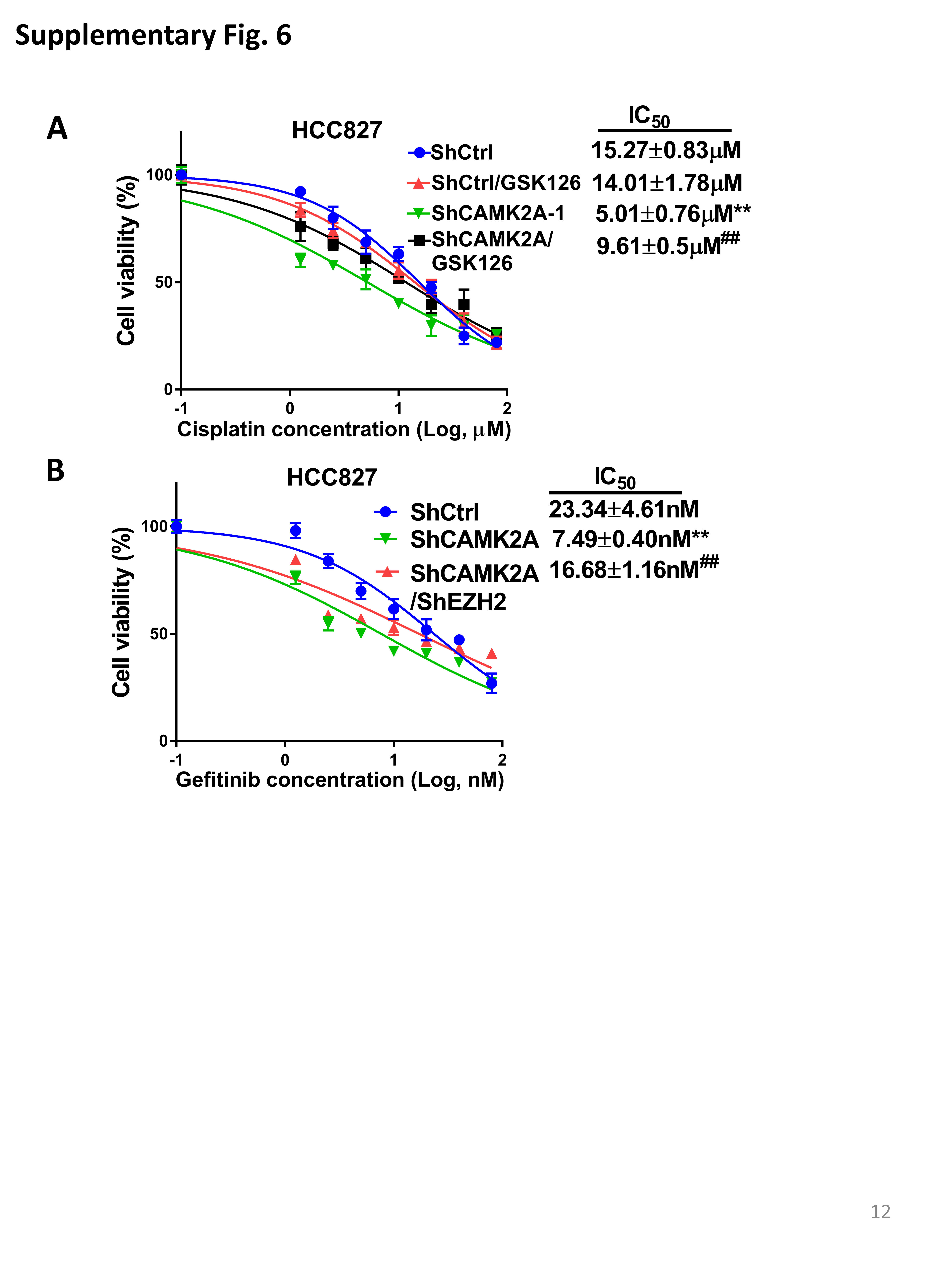

Supplement: Supplementary file 7 — Supplementary Figure 6 [file 41419_2020_2553_MOESM7_ESM.tif]

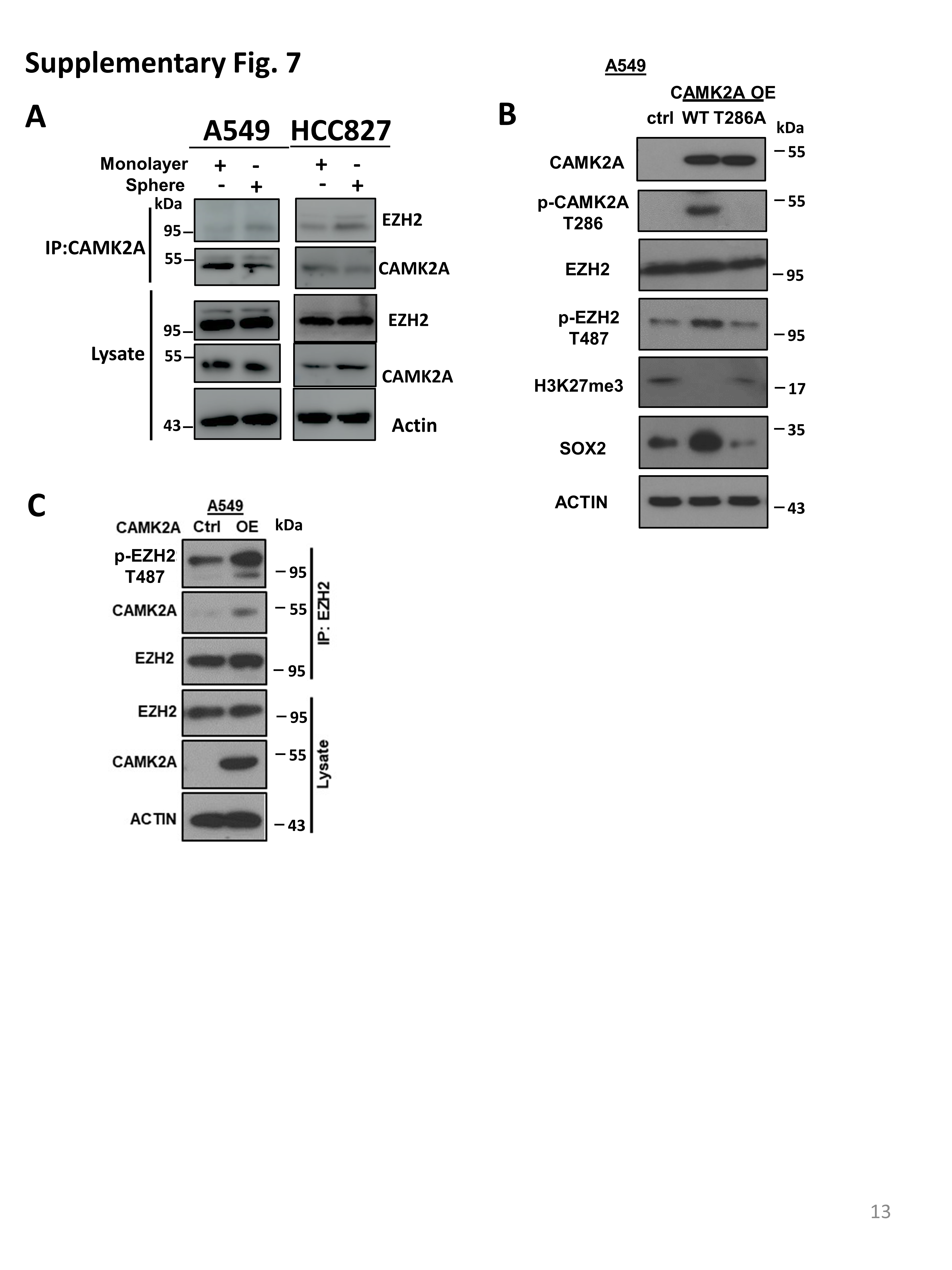

Supplement: Supplementary file 8 — Supplementary Figure 7 [file 41419_2020_2553_MOESM8_ESM.tif]

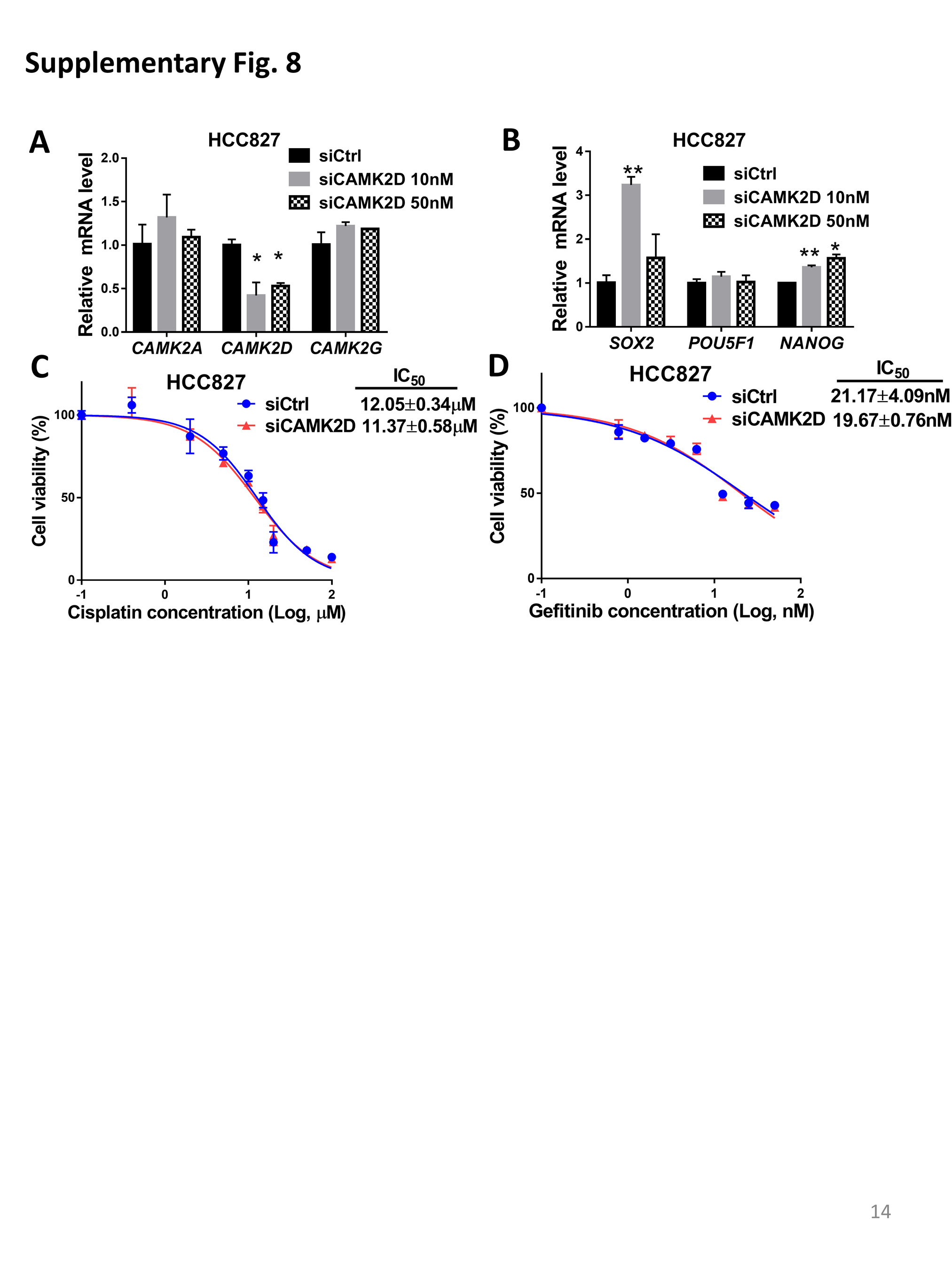

Supplement: Supplementary file 9 — Supplementary Figure 8 [file 41419_2020_2553_MOESM9_ESM.tif]
