# Supplementary material for: CAMK2A supported tumor initiating cells of lung adenocarcinoma by upregulating SOX2 through EZH2 phosphorylation
Source: Cell Death Dis. 2020 Jun 1;11(6):410. doi: 10.1038/s41419-020-2553-6 (PMC7264342; doi:10.1038/s41419-020-2553-6)
Supplement: Supplementary file 10 — Supplementary figure legends [file 41419_2020_2553_MOESM10_ESM.docx]

**Supplementary figure legends**

**Supplementary Figure 1.** Analysis of effects of CAMK2A expression on survival. (A, B) IHC evaluation of CAMK2A expression in lung AD showing high (A), and low expression (B), respectively. (C) Kaplan Meier analysis of recurrent free survival of AD stratified by CAMK2A expression. (D) Correlation analysis between CAMK2A and p-CAMK2A T286 expression in 268 primary resected lung AD by immunohistochemistry.

**Supplementary Figure 2.** Analysis of effects of CAMK subunits expression on survival. (A, B) COX regression analysis of CAMK2B, D and G expressions on Regression Free Survival (RFS) (A) and Overall survival (OS) (B) using expression and survival data from public database ([www.kmplot.com/lung](http://www.kmplot.com/lung))

**Supplementary Figure 3.** Effects of CAMK2A on TIC properties in lung AD cells. (A) CAMK2A expression in lung cancer cell lines by immunoblot. (B) Expression of p-CAMK2A T286 in CAMK2A knockdown HCC827 and PDCL#24 cells by immunoblot. (C) Knockdown of CAMK2A attenuated tumor growth from HCC827 cells-derived xenograft. (D) Expression of p-CAMK2A T286 in CAMK2A-overexpressing H1299 and A549 cells by immunoblot. (E) CAMK2A overexpression facilitated tumor growth in H1299-derived xenografts. **, p < 0.01; ***, p < 0.001 compared with control. Data represented mean ± SD.

**Supplementary Figure 4.** Effects of CAMK2A on responses of lung AD cells to targeted therapy and cytotoxic drugs. (A) Cell viability of HCC827 GR and parental cells under gefitinib treatment. (B) Cell viability of A549 CR and parental cells under cisplatin treatment. (C, D) Sphere formation ability was increased in HCC827GR (C) and A549CR (D) cells compared to corresponding parental cells. (E) Effect of KN93 on viability of A549 cells compared to KN92 control. (F) Treatment of KN93 suppressed expression level of p-CAMK2A T286 in HCC827 cells. (G) Cell viability of HCC827 cells cotreated with gefitinib and KN93 compared to KN92. (H) Cell viability of A549 cells cotreated with cisplatin and KN93 compared to KN92. *, p < 0.05; **, p < 0.01; compared with control, Data represented mean ± SD.

**Supplementary Figure 5.** Effects of CAMK2A on expression of histone marks*.* (A) Expression of H3K4me3 in CAMK2A-manipulated cells by immunoblot. (B) Expression of H3K27ac in CAMK2A-manipulated cells by immunoblot. (C) Expression of H3K9me2 in CAMK2A-manipulated cells by immunoblot.

**Supplementary Figure 6.** Effect of EZH2 inhibition on CAMK2A-mediated drug resistance (A) EZH2 inhibition by GSK-126 increased cisplatin sensitivity of CAMK2A-knockdown HCC827 cells by MTT assay. (B) EZH2 knockdown increased gefitinib sensitivity of CAMK2A-knockdown HCC827 cells. **, p < 0.01, compared with scramble control, ^##^, p<0.01 compared to shCAMK2A. Data represented mean ± SD.

**Supplementary Figure 7.** CAMK2A phosphorylated EZH2 at T487. (A)Immunoprecipitated EZH2 level by CAMK2A antibody in tumorspheres and monolayers by immunoblot. (B) Expression level of CAMK2A, p-CAMK2A T487, EZH2, p-EZH2 T487, SOX2 and H3K27me3 in A549 cells with CAMK2A- or CAMK2A T286A-overexpression. (C) Immunoprecipitated p-EZH2 T487 level by EZH2 antibodies in A549 cells with or without CAMK2A overexpression.

**Supplementary Figure 8.** (A) Expression level of CAMK2 subunits in HCC827 cells with or without siCAMK2D KD by q-PCR. (B) Expression level of *SOX2*, *POUSF1* and *NANOG* in HCC827 cells with or without siCAMK2D KD by q-PCR. (C, D) Cell viability of HCC827 cells with or without CAMK2D KD under treatment with cisplatin (C) and gefitinib (D).
